# Human Plasma-Derived 3D Cultures Model Breast Cancer Treatment Responses and Predict Clinically Effective Drug Treatment Concentrations

**DOI:** 10.3390/cancers12071722

**Published:** 2020-06-29

**Authors:** Kristin Calar, Simona Plesselova, Somshuvra Bhattacharya, Megan Jorgensen, Pilar de la Puente

**Affiliations:** 1 Cancer Biology and Immunotherapies Group, Sanford Research, Sioux Falls, SD 57104, USA; kristin.calar@sanfordhealth.org (K.C.); somshuvra.bhattacharya@sanfordhealth.org (S.B.); megan.jorgensen@sanfordhealth.org (M.J.); 2 Biochemistry and Molecular Biology II, University of Granada, 18071 Granada, Spain; splessel@ugr.es; 3 MD/PhD Program, University of South Dakota Sanford School of Medicine, Sioux Falls, SD 57105, USA; 4 Department of Surgery, University of South Dakota Sanford School of Medicine, Sioux Falls, SD 57105, USA; 5 Flow Cytometry Core, Sanford Research, Sioux Falls, SD 57104, USA

**Keywords:** 3D culture, breast cancer, treatment response, precision medicine, drug metrics

## Abstract

Lack of efficacy and a low overall success rate of phase I-II clinical trials are the most common failures when it comes to advancing cancer treatment. Current drug sensitivity screenings present several challenges including differences in cell growth rates, the inconsistent use of drug metrics, and the lack of translatability. Here, we present a patient-derived 3D culture model to overcome these limitations in breast cancer (BCa). The human plasma-derived 3D culture model (HuP3D) utilizes patient plasma as the matrix, where BCa cell lines and primary BCa biopsies were grown and screened for drug treatments. Several drug metrics were evaluated from relative cell count and growth rate curves. Correlations between HuP3D metrics, established preclinical models, and clinical effective concentrations in patients were determined. HuP3D efficiently supported the growth and expansion of BCa cell lines and primary breast cancer tumors as both organoids and single cells. Significant and strong correlations between clinical effective concentrations in patients were found for eight out of ten metrics for HuP3D, while a very poor positive correlation and a moderate correlation was found for 2D models and other 3D models, respectively. HuP3D is a feasible and efficacious platform for supporting the growth and expansion of BCa, allowing high-throughput drug screening and predicting clinically effective therapies better than current preclinical models.

## 1. Introduction

Breast cancer (BCa) is a heterogeneous disease, which includes different biological entities characterized by distinct clinical behaviors and responses to treatment [1,2,3]. Most BCa deaths, around 90%, can be contributed to recurrence or the spread of the disease [4,5]. Nonetheless, the fact that the BCa population is highly heterogeneous is generally overlooked and most BCa patients still receive the same treatment [6,7,8]. Despite significant improvements in research and development in the cancer field, about 80% of oncology drugs in clinical trials fail to receive U.S. Food and Drug Administration (FDA) approval [9,10,11]. Recent studies have found the overall success rate of phase I-II clinical trials for cancer treatment to be extremely low (3.4%) [11], and the lack of efficacy was found to be the most common reason for failure (>50%) over other factors such as safety, study design or data analysis [12,13]. These studies clearly indicate the need for more suitable preclinical models that would provide an accurate prediction of clinical efficacy. Unfortunately, the most common preclinical cancer models do not deliver full fidelity of the heterogeneous BCa tumors in the context of a tumor-like environment. Animal models, including patient-derived xenografts (PDX), are very useful in the recreation of this environment, but they are costly, relatively time consuming, and their reproducibility and translatability to human cancer clinical trials is very low [14,15]. Traditional two-dimensional (2D) models do not adequately mimic cellular interactions and fail to adequately recapitulate the in vivo-like architecture, directly influencing diffusion capabilities and drug resistance [16]. Recent studies have indicated that the recapitulation of the cell-cell interactions, the recreation of the extracellular matrix (ECM) architecture, the simulation of drug transport and growth factors, and the amenability to high-content screening are a few well-established advantages of three-dimensional (3D) cell co-culture models [17,18]. In addition, preclinical models that solely use cancer cells as monocultures frequently lead to disappointing therapeutic outcomes due to the fact that environment-mediated drug resistance is a major contributor to lack of therapeutic efficacy [19,20,21,22,23,24,25]. All this evidence suggests that 3D cell culture techniques provide useful advantages for representing in vivo tumor-like environments and modelling drug sensitivity [26,27,28]; however, there are still challenges. Some of the current limitations to these 3D culture models include the incorporation of exogenous ECM materials that might introduce undesired confounding artifacts to the recreation of tumor behaviors, the establishment of differences in cell growth rates in the model, and the varying use of drug metrics across different culture techniques [29]. Protein-based hydrogels, such as fibrin hydrogels, have been used in a wide range of applications including regenerative medicine, drug delivery, and as 3D culture models [30,31]. Fibrin scaffolds created through the cross-linking of commercial fibrinogen with thrombin have reported success in cancer growth and drug screening applications in different cancer types, including breast cancer [31,32]. Here, we present a physiological, patient-derived, tumor-like 3D culture method that utilizes patient plasma (fibrinogen cross-linking to fibrin) as the matrix, referred to as the human plasma-derived 3D culture model (HuP3D), which supports the efficient growth and expansion of primary breast cancer tumors, as well as allows for the screening of cancer drug responses in BCa cell lines and primary BCa tumors through several drug metrics for further prediction of clinical therapeutic efficacy.

## 2. Results

### 2.1. Chemical and Physical Characterization of the HuP3D Culture Model

**Figure 1:**
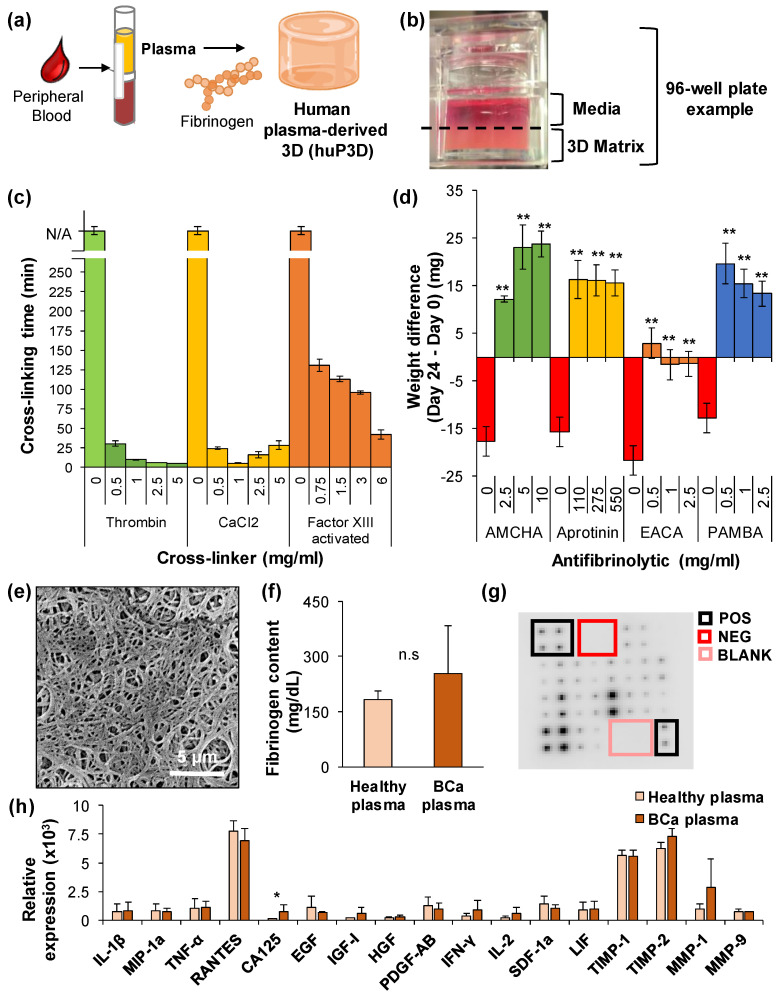
Chemical and physical characterization of the human plasma-derived 3D (HuP3D) culture model. (**a**) HuP3D matrices are formed through the cross-linking of fibrinogen, found naturally in plasma, into fibrin. These matrices can include cells either from cell lines or tissue biopsies. (**b**) HuP3D cultures in 96-well plates generate a 3 mm tall gelatinous-like scaffold matrix where media is added on top to overcome drying. (**c**) Optimization cross-linking studies. A measurement of the time (minutes) to achieve matrix cross-linking using three relevant cross-linking agents of the blood coagulation process including thrombin (0–5 mg/mL), CaCl_2_ (0–5 mg/mL), and factor XIII (0–6 mg/mL) (mean ± SD, *n* = 3). (**d**) Optimization stabilization studies. Stabilization effect studies of preventing fibrin degradation and stability improvement in the scaffold were achieved by testing several chemical antifibrinolytic agents including trans-4-(aminomethyl) cyclohexane carboxylic acid (AMCHA) (0–10 mg/mL), aprotinin (0–550 mg/mL), epsilon-aminocaproic acid (EACA) (0–2.5 mg/mL), and 4-(aminomethyl)benzoic acid (PAMBA) (0–2.5 mg/mL) (mean ± SD, *n* = 3). Scaffold stability was studied by measuring each scaffold weight at day 0 and at the conclusion of a three-week time period. ** *p* < 0.001 compared to lack of stabilizer. (**e**) Representative SEM micrograph of an acellular HuP3D scaffold cultured for 4 days. Scale bar: 5 µm. (**f**) Fibrinogen levels (mg/dL) present in plasma from healthy subjects (mean ± SD, *n* = 5) and breast cancer (BCa) patients (mean ± SD, *n* = 2) used in the included studies. (**g**) Example of the custom human cytokine array. (**h**) Relative protein expression of HuP3D cultures made of plasma from healthy subjects and BCa patients (mean ± SD, *n* = 2). * *p* < 0.05.

Human plasma-derived 3D culture (HuP3D) models were created by cross-linking fibrinogen, a blood plasma protein responsible for normal blood clotting when converted into fibrin (Figure 1a) [33], generating a gelatinous-like scaffold matrix using traditional tissue culture surfaces as the recipient mold, with media added on top to overcome drying of the matrix (Figure 1b). To optimize conditions for cell culture, we needed a stable 3D matrix with fast, yet controlled, cross-linking capabilities and a porous intrinsic structure. For that purpose, three classical cross-linkers were tested to determine which component would produce optimal cross-linking of the HuP3D. Plasma requires the presence of a cross-linking agent in order to form a 3D scaffold matrix, otherwise, it remains in a liquid form with no reportable cross-linking time (represented as not applicable, N/A) when no cross-linking agent is added. The addition of thrombin allowed the cross-linking time to be reduced with increasing concentrations to a value of 5 min at 5 mg/mL. Adding CaCl_2_ generated the fastest cross-linking time (4 min) at a concentration of 1 mg/mL, and increasing concentrations proved to be less efficacious. Factor XIII required activation by incorporating calcium, and the fastest cross-linking time for this component was over 40 min at a concentration of 6 mg/mL (Figure 1c). With this data, CaCl_2_ at a 1 mg/mL concentration was determined to be the optimal concentration for cross-linking for the remainder of the experiments. Another important aspect to consider is that fibrin clots tend to degrade or lyse overtime [34], so in order to reduce HuP3D degradation, as well as maintain structural integrity and stability, various antifibrinolytics were tested. HuP3D integrity and stability was measured after 24 days in culture by comparing the weight of the 3D cultures at day 24 to the weight of the 3D cultures at day 0. A lack of antifibrinolytics incorporated into the matrix resulted in a weight loss of about 16.98 ± 3.77 mg (representing around 5% to 9% loss of total weight). While epsilon-aminocaproic acid (EACA) was not able to sustain an integrity benefit at the concentrations tested, the other three antifibrinolytics resulted in weight gains of at least 5 to 10% of their total weight (Figure 1d). In particular, trans-4-(aminomethyl)cyclohexanecarboxylic acid (AMCHA) at 5 and 10 mg/mL resulted in the highest weight gain of about 24 mg (representing around 10% gain of total weight), and this was defined to be the recommended concentration for all remaining experiments. Working under the recommended cross-linker and stabilizer concentrations, scanning electron microscopy (SEM) was used to determine the physical structure of HuP3D cultures. SEM (Figure 1e) images revealed a porous structure with a network of interconnecting fibers, which will aid in gas diffusion, nutrient supply, and waste removal through the 3D culture matrix [35,36]. Fibrinogen levels were found to be non-significantly different between plasma from healthy subjects and BCa patients (Figure 1f). In addition, plasma from healthy subjects and BCa patients was cross-linked to generate HuP3D cultures, which were further characterized by the cytokine milieu of the 3D culture matrix. Using a custom antibody array, we measured proteins (in duplicate) at the baseline, day 0, of acellular HuP3D cultures in serum free media (Figure 1g). Relative protein expression was compared for healthy subjects and BCa patients and no significant differences were found, except for a higher expression of CA125, which is a common blood test marker for BCa detection (Figure 1h). High levels of RANTES, TIMP-1, and TIMP-2 were present in both groups of plasma, as well as detectable levels of IL-β1, MIP-1a, TNFα, SDF-1, MMP1, MMP9, INF-γ, IL-2 LIF, EGF, IGF-I, HGF, and PDGF-AB. 

### 2.2. HuP3D Culture Supports BCa Proliferation 

**Figure 2:**
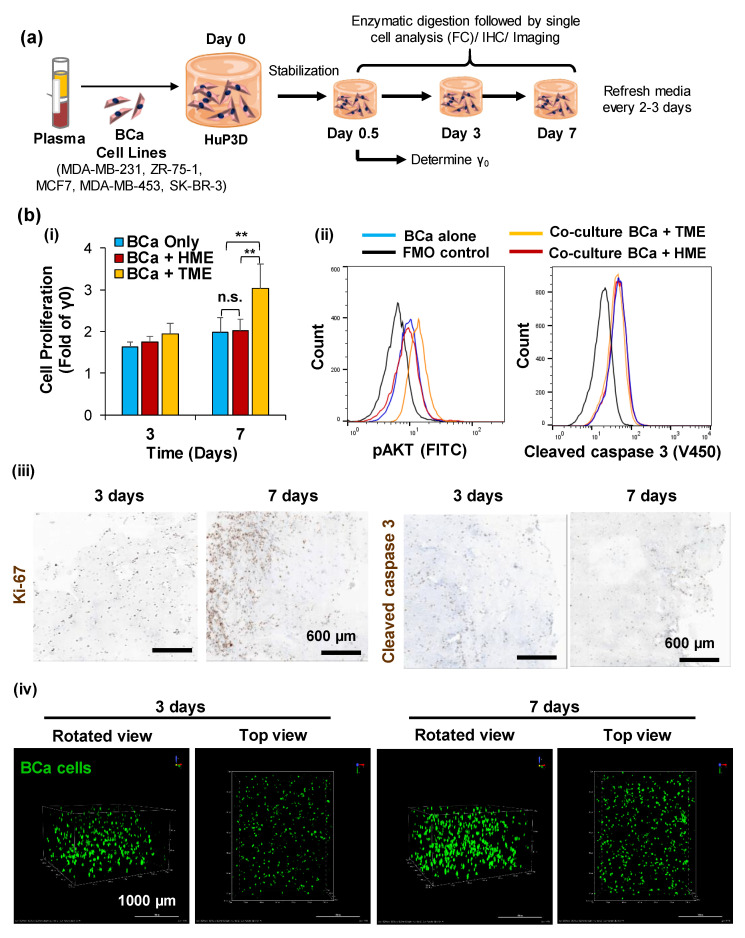
HuP3D cultures allow BCa cell proliferation. (**a**) HuP3D matrices are created through the cross-linking of plasma fibrinogen and the matrices can include pre-labeled BCa cells from 5 cell lines representing different BCa subtypes in culture with numerous variations of microenvironment components. These matrices were cultured for 0.5, 3, and 7 days followed by enzymatic digestion and single cell analysis using flow cytometry, immunohistochemistry (IHC), or confocal imaging. (**b**) Cell proliferation and apoptosis of the five BCa cell lines either alone, in co-culture with a healthy microenvironment (HME), or in co-culture with a tumor microenvironment (TME) in the HuP3D matrix presented as (**i**) cell fold of γ_0_ for 3 and 7 days (mean ± SD, *n* = 4); (**ii**) representative flow cytometry histograms of FITC-pAKT and V450-cleaved caspase 3 signals compared to the fluorescence minus one (FMO) control at day 7 for the MDA-MB-231 BCa cell line (mean ± SD, *n* = 3); (**iii**) representative IHC images of the MDA-MB-231 cell line for Ki67 and caspase 3 staining at days 3 and 7, scale bar = 600 µm; (**iv**) representative confocal images on day 3 and day 7 to monitor proliferation of the MDA-MB-231 BCa cell line grown within the HuP3D (DiO: green). Scale bar = 1000 µm. ** *p* < 0.001, n.s. not significant.

Human plasma from healthy subjects was used when BCa cell lines were integrated into HuP3D cultures, merely due to the lack of access to matching plasma from the BCa patients from which the cell lines were derived and in order to develop a culture technique amenable to utilization by a wide range of different research laboratories. Five BCa lines (Table 1) were incorporated into HuP3D cultures where, after initial stabilization of the cells within the matrix for half a day, media was added on top and refreshed every 2–3 days over the course of the experiment. Proliferation assays were performed at days 0.5, 3, and 7 (Figure 2a). In particular, BCa cell lines were cultured alone (BCa only), in combination with a healthy microenvironment (HME) derived from healthy breast tissue, or in combination with a tumor microenvironment (TME) containing accessory cells derived from BCa tumor biopsies after sorting out CD44+ BCa cells. The five BCa cell lines alone showed very similar results in proliferation with an increased proliferation of approximately 1.6-fold and 2-fold compared to γ_0_ at day 3 and 7, respectively. While co-culture with a HME did not improve BCa proliferation, co-culture with a TME at day 7 significantly increased cell proliferation to 3-fold in all the BCa cell lines tested, reflecting the important role of the TME on tumor proliferation (Figure 2b(i), Figure S1a and Figure S2). With this data we further corroborated that co-culture with a TME increased the expression of proteins involved in survival and proliferation (pAKT), while no effect was found in apoptotic pathways (cleaved caspase 3) at the single cell level focusing on the BCa cell population, using flow cytometry (Figure 2b(ii)). We further confirmed these results using immunohistochemistry IHC, which revealed an increased proliferation through pixel count, of an increased Ki67 expression over time, at day 7, while apoptosis expression, measured by cleaved caspase 3, remained unaltered (Figure 2b(iii)). Moreover, we evaluated HuP3D cultures using confocal imaging (Figure 2b(iv)). HuP3D cultures revealed a significant increase in the number of BCa cells (DiO labeled) and increased clustering capabilities at day 7 compared to day 3 (Figure S1a,b).

### 2.3. HuP3D Culture Enhances Extracellular Vesicle (EV) Secretion 

**Figure 3:**
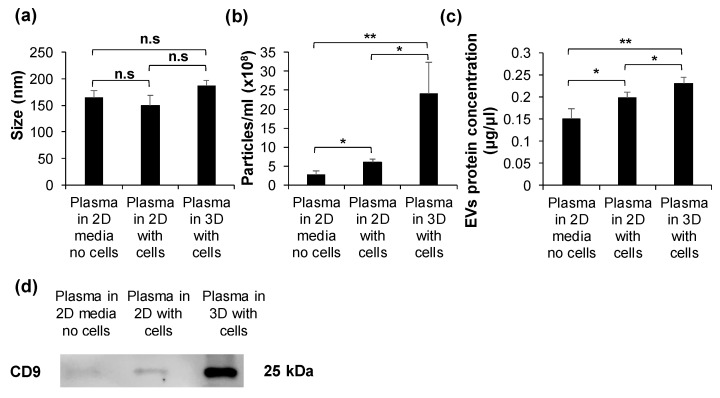
Extracellular vesicle production in the HuP3D matrix increases in comparison to 2D cultures with and without cells from the MDA-MB-231 BCa cell line. (**a**) Characterization of the extracellular vesicles was completed using dynamic light scattering (DLS) analysis for size (nm) (mean ± SD, *n* = 3). (**b**) EV particle concentration/mL (mean ± SD, *n* = 3). (**c**) Protein concentration (μg/μL) using a BCA protein assay kit (mean ± SD, *n* = 4). (**d**) Western blot analysis on the expression of the EV marker CD9. * *p* < 0.05 ** *p* < 0.001, n.s. not significant.

HuP3D cultures are formed with human plasma, which contains extracellular vesicles (EVs). We therefore sought to study the amount of EVs incorporated from the plasma itself and the de novo secretion of EVs by the cells in 2D and HuP3D cultures. Human plasma from each subject was separated proportionally to incorporate the same amount of plasma into each culture condition. Conditions include plasma in culture media with no cells, plasma instead of fetal bovine serum (FBS) in culture media with cells in 2D, and plasma in HuP3D (matrix and culture media) (*n* = 3). EVs showed a consistent size of around 150 nm for all three culture conditions (Figure 3a). The number of particles/mL detected using dynamic light scattering (DLS) revealed a significant 6-fold increase in EVs in HuP3D cultures compared to 2D cultures in plasma incubated with the same amount of BCa cells. We also found that the EV particles/mL baseline control of just plasma in media with no cells was increased by about 2-fold and 10-fold in 2D and HuP3D cultures, respectively, revealing the additional EV secretion by the BCa cells (Figure 3b). We further confirmed that the exosome protein concentration was boosted in HuP3D cultures compared to the baseline plasma media and 2D cultures through a BCA protein assay. While baseline plasma in media and no cells showed 0.14 µg/µL EVs, 2D and HuP3D cultures showed a significant increase of about 1.3-fold and 1.53-fold, respectively (Figure 3c). Finally, we confirmed the expression of a common EV marker, CD9, through western immunoblotting to further validate the presence and enhancement of EVs in HuP3D cultures compared to 2D (Figure 3d).

### 2.4. HuP3D Cultures Allow High-Throughput Drug Screening of BCa Cell Lines 

The five BCa cell lines in HuP3D were screened for seven common BCa chemotherapeutics (capecitabine (CAP), cyclophosphamide (CYCLO), docetaxel (DTX), epirubicin (EPI), methotrexate (MTX), paclitaxel (PTX), and carboplatin (CARBO)) at nine increasing drug concentrations (0.1 nM–300 µM) plus a dimethyl sulfoxide (DMSO) control (γ_ctrl_) and analyzed using flow cytometry at days 0.5 and 7. Data obtained was used to calculate 10 relevant drug metrics based on relative cell count and growth rate (GR) value curves according to previously described methods, using the online GR calculator tool developed by Hafner et al. [37,38,39] (Figure S3b). Briefly, the HuP3D culture drug metrics from the relative cell count curve included the half maximum inhibitory concentration (IC_50_), maximal measured efficacy (E_max_), area under the curve (AUC), half maximal response concentration (EC_50_), and the effect of the drug at the infinite concentration (E_inf_). HuP3D culture drug metrics from the GR curve included the half maximum growth rate inhibition concentration (GR_50_), the maximum effect of the drug at the highest tested concentration (GR_max_), area over the curve (GR_AOC_), the half maximal growth rate inhibition response concentration (GEC_50_), and the growth rate inhibition effect of the drug at the infinite concentration (GR_inf_). Figure S3b shows the changes in all the metrics when comparing cytostatic and cytotoxic drugs.

**Figure 4:**
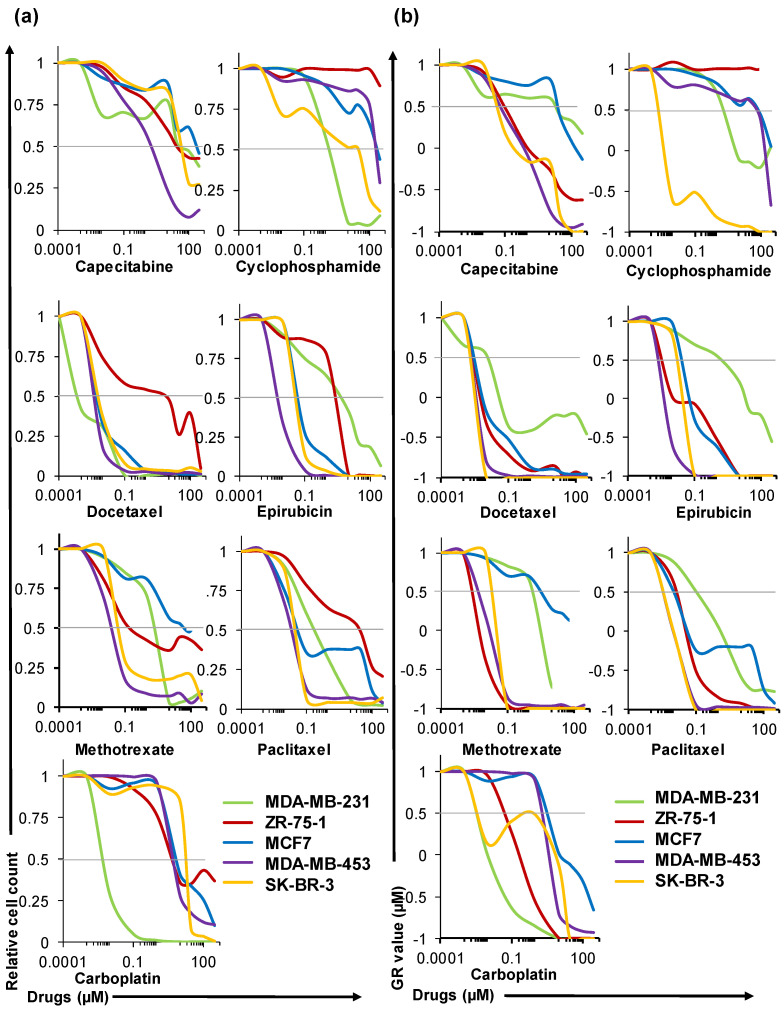
HuP3D cultures, created with healthy fibrin plasma, allow for high-throughput drug screening for BCa cell lines. (**a**) Results showing the effect of increasing concentrations (0.0001, 0.001, 0.01, 0.1, 1, 10, 30, 100, and 300 µM) of capecitabine (CAP), cyclophosphamide monohydrate (CYCLO), docetaxel (DTX), epirubicin hydrochloride (EPI), methotrexate (MTX), paclitaxel (PTX), and carboplatin (CARBO) on 5 BCa cell lines when grown in HuP3D cultures on BCa survival (mean, *n* = 4) and (**b**) on GR values (mean, *n* = 4).

BCa cell lines after treatment were identified as DiD+, with viability confirmed using Sytox green viability marker, and analyzed using flow cytometry, where the data was normalized against an internal counting bead control. Relative cell count (Figure 4a) and GR value (Figure 4b) curves for all the screened conditions revealed heterogeneous therapeutic responses. For example, while docetaxel and paclitaxel were cytotoxic over four of the BCa cell lines in the relative cell count curve, a milder cytostatic effect was found in ZR-75-1. However, in the GR curve for docetaxel and paclitaxel, the cytostatic effect was exhibited only in the MDA-MB-231 cell line. Epirubicin metrics were consistent in cell count and GR curves, which exhibited MDA-MB-231 as the most resistant cell line. Capecitabine was revealed as a cytostatic drug over the concentrations tested, and cyclophosphamide showed ZR-75-1 as resistant at all doses, even at the higher doses tested. Methotrexate and carboplatin also showed a heterogeneous response among the BCa cell lines with MDA-MB-231 being the most sensitive to carboplatin and MCF7 being the most resistant to methotrexate.

**Figure 5:**
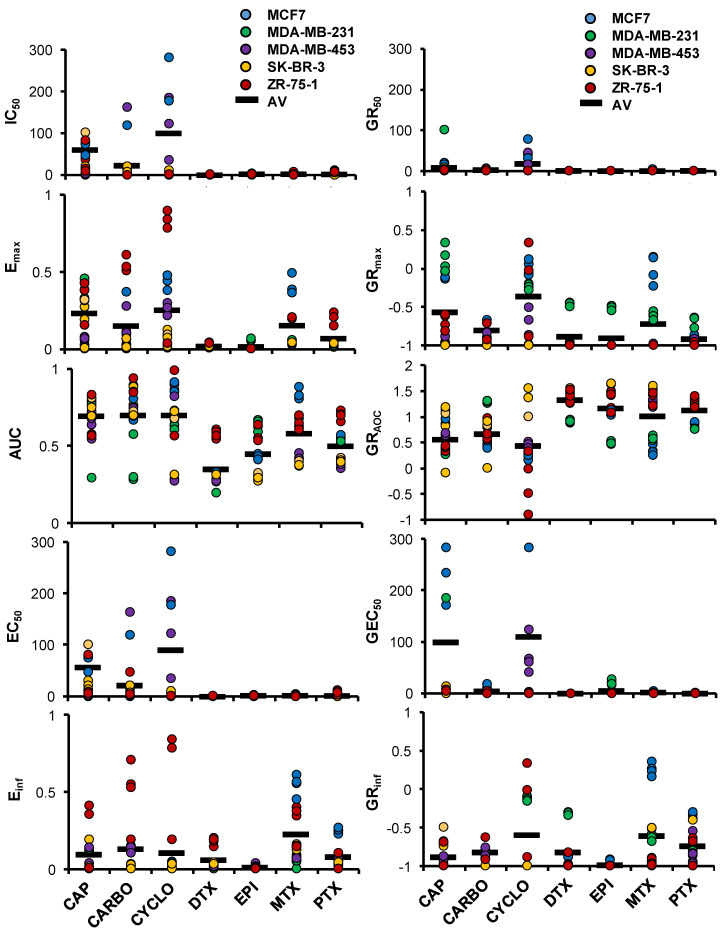
HuP3D drug metrics reveal heterogeneous responses. Results showing IC_50_, E_max_, AUC, EC_50_, E_inf_, GR_50_, GR_max_, GR_AOC_, GEC_50_, and GR_inf_ values in HuP3D cultures for 5 BCa cell lines (*n* = 4/cell line) and the mean average for the 5 cell lines for capecitabine (CAP), carboplatin (CARBO), cyclophosphamide monohydrate (CYCLO), docetaxel (DTX), epirubicin hydrochloride (EPI), methotrexate (MTX), and paclitaxel (PTX).

Using the GR calculator tool, multiple different drug metrics were calculated (Table S1) and all drug metrics revealed heterogeneity among the BCa cell lines, drugs, and even between the metrics themselves. For metrics measuring concentrations (IC_50_, EC_50_, GR_50_, and GEC_50_), the *y*-axis scale goes from 0.1 nM to 300 µM, whereas GR_max_ and GR_inf_ values lie between 1 and −1 (Figure 5). While IC_50_, EC_50_, and their counterparts GR_50_ and GEC_50_ showed that DTX, EPI, MTX, and PTX were very effective at low doses for the 5 cell lines, all metrics for CYCLO revealed a large variability of therapeutic response amongst the different cell lines. E_max_ and E_inf_ showed ZR-75-1 as one of the most resistant cell lines to all therapies, followed by MCF7. All these results clearly resonate with the necessity of having multiple drug metrics to predict therapeutic efficacy, and the critical need for personalized approaches allowing for the prediction of response to therapy in a precision-based manner.

### 2.5. HuP3D Culture Drug Metrics Correlate Better Than Other In Vitro Models with Clinical Data 

**Figure 6:**
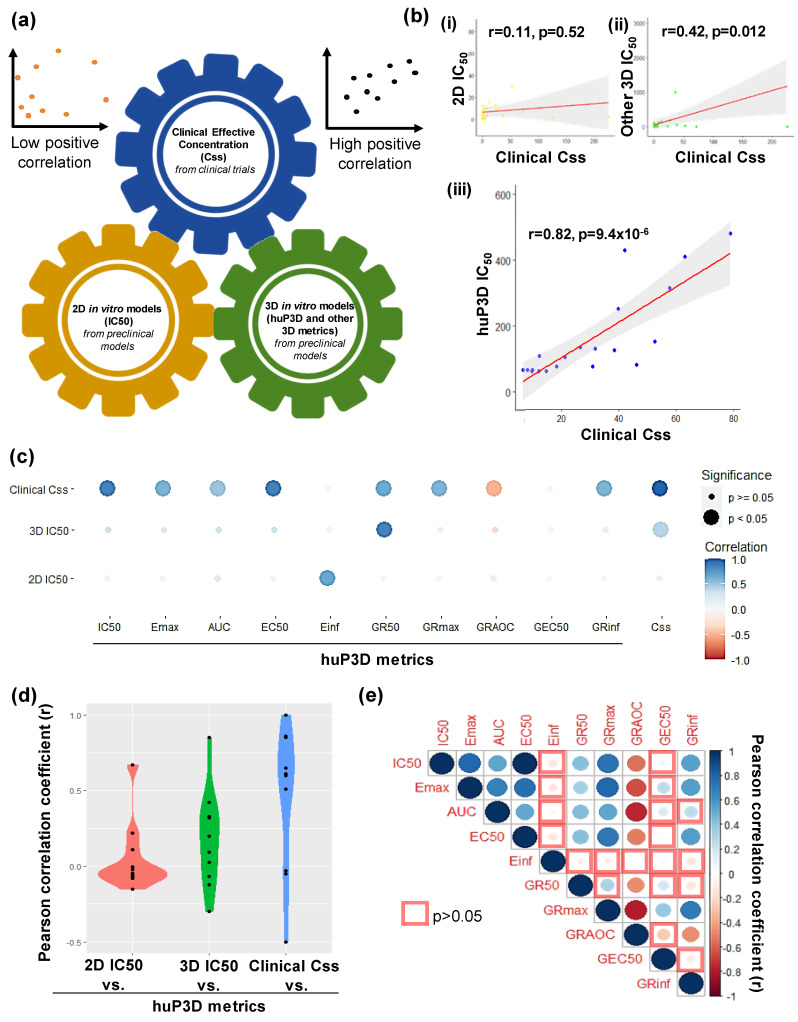
HuP3D culture drug metrics correlate better than other in vitro models with clinical data. (**a**) A representative cartoon of the correlation studies using literature search metrics from 2D models (IC_50_), literature search metrics from other 3D models (IC_50_), and HuP3D metrics, as well as effective concentrations in patients from phase I or II studies that examined the pharmacokinetics of the tested chemotherapies (steady state concentration, Css) and examples of low positive and high positive correlations. (**b**) Pearson correlation (r) and *p* significance values of (**i**) literature 2D IC_50_ and clinical Css; (**ii**) literature 3D IC_50_ for other 3D models and clinical Css; (**iii**) HuP3D IC_50_ and clinical Css. (**c**) Dot plot visualization of the Pearson correlation (r) and *p* significance values generated in Seurat. Color of the dot indicates the Pearson correlation of HuP3D metrics and clinical Css, other 3D IC_50_, and 2D IC_50_, and the size indicates the significance of the correlation. (**d**) Violin plot of the Pearson correlation coefficient (r) values of HuP3D metrics compared to 2D IC_50_, other 3D IC_50_, and clinical Css. (**e**) Correlogram showing the Pearson correlation coefficient (r) values for IC_50_, E_max_, AUC, EC_50_, E_inf_, GR_50_, GR_max_, GR_AOC_, GEC_50_, and GR_inf_ metrics in HuP3D cultures.

While establishing drug response metrics in vitro is very useful for the discovery and repurposing of drugs, the validation of those drug response metrics as predictive tools of clinically effective treatments in patients would be far superior. In order to assess the predictive value of the drug response metrics obtained in our previous assays, we compared them with metric data obtained from relevant literature for 2D models (IC_50_) and other 3D models (IC_50_), as well as effective concentrations in patients from phase I or II studies that examined the pharmacokinetics of the tested chemotherapies (steady state concentration, Css) (Figure 6a). A scatterplot correlation graph allowed us to establish the strength, direction, and form of the relationship between the in vitro models and the Css clinical data, with Pearson correlation coefficients (r) that were calculated to measure the strength of those relationships. While a very weak positive correlation (r = 0.11) existed for the comparison of 2D IC_50_ to clinical Css values (Figure 6b(i)), moderate (r = 0.42) to strong (r = 0.82) correlations were revealed for IC_50_ values of other 3D models (Figure 6b(ii)) and the HuP3D culture model (Figure 6b(iii)) compared to the clinical Css, respectively. When Pearson correlation coefficients of the 10 HuP3D metrics where compared to the clinical Css, a significant and strong correlation (higher than 0.5) was found for 8 of these metrics (Figure 6c). Only the GR_50_ and E_inf_ of the HuP3D metrics significantly and strongly correlated with the IC_50_ of other 2D and 3D models, respectively (Figure 6c). In addition, a violin plot clearly shows how most of the HuP3D metrics correlated well with clinical Css values (Figure 6d), from purely a distribution standpoint, revealing the potential of some of the drug metrics for HuP3D to potentially evaluate clinically effective therapeutic responses relevant to patients. Not surprisingly, HuP3D metrics correlate moderately with other 3D model metrics and poorly with 2D model metrics (Figure 6d).

To further assess the inter-correlation among the 10 HuP3D metrics, a correlogram was used to depict the Pearson correlation coefficients (Figure 6e). IC_50_ and EC_50_ showed the strongest positive correlation among all the compared metrics. Metrics derived from the relative cell count curve (IC_50_, E_max_, AUC, EC_50_), except E_inf_, showed a significant and strong positive correlation between them. GR_50_ and GR_max_ also positively correlated with the metrics derived from the relative cell count curve, except E_inf_. On the other hand, GR_AOC_ significantly and strongly negatively correlated with all the metrics, except E_inf_ and GEC_50_.

### 2.6. HuP3D Cultures Support Primary BCa Proliferation and Retrospective Prediction of Therapeutic Efficacy in BCa Patients

**Figure 7:**
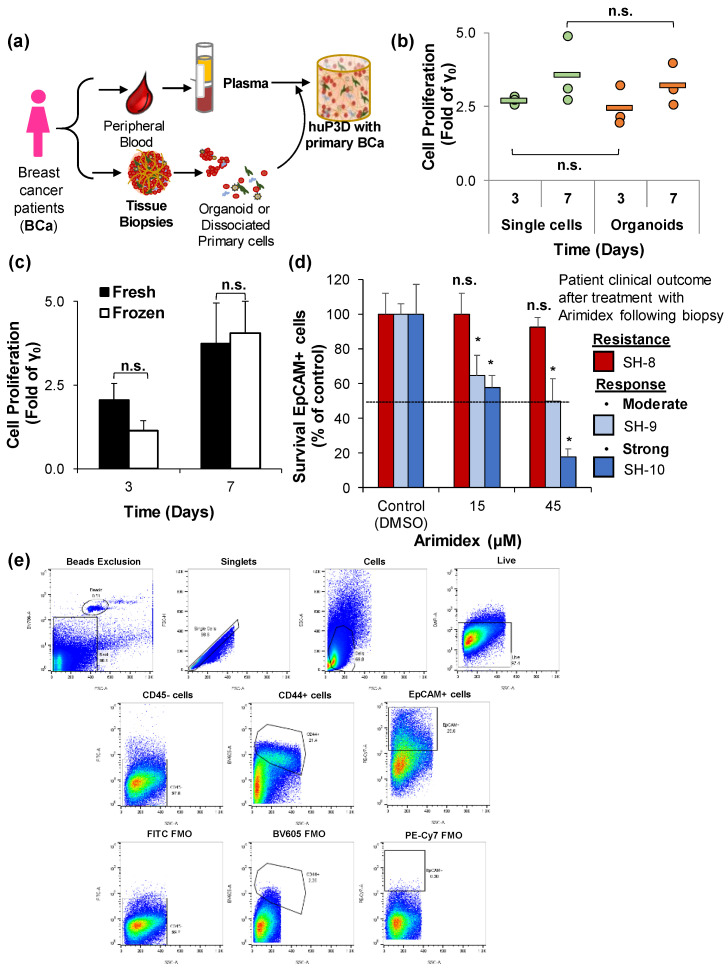
HuP3D cultures promote growth of patient biopsy material (fresh or frozen) and recreate therapeutic responses shown in patients in an in vitro environment. (**a**) Patient biopsies and blood samples were obtained from BCa cancer patients. Tissue biopsies were either enzymatically digested into single cells or processed into small organoid tissue sections. Both tissue processing methods (single cells and organoids) were grown in HuP3D cultures made from the matching patient plasma. (**b**) Cell proliferation in HuP3D cultures that have been cultured for 3 and 7 days, shown as fold of γ_0_, either as single cells or small organoids, n.s., not significant (mean ± SD, *n* = 3). (**c**) Cell proliferation in HuP3D cultures that have been cultured for 3 and 7 days, shown as fold of γ_0_, either as fresh cells or as the same cells subjected to a freeze/thaw cycle (frozen), n.s., not significant (mean ± SD, *n* = 3); (**d**) Effect of increasing concentrations of Arimidex (7 days) on primary BCa EpCAM+ cell survival in HuP3D cultures, (*) *p* < 0.05 compared to control, n.s., not significant (mean ± SD, *n* = 3). (**e**) Gating strategy for analysis of patient biopsy material grown in HuP3D cultures. Data acquisition was completed by collecting information for a specified number of events determined by counting beads. Firstly, cellular populations were isolated from beads then singlets were gated. Cell populations were further selected and the live cell population identified by live/dead viability marker. Following this, CD45- FITC cells, EpCAM+ (CD326+) PeCy7 cells, and BV605 (CD44+) cells were identified from the live cell population. Fluorescence minus one (FMO) controls were used to set the gating for each population FITC (CD45), PeCy7 (EpCAM), and BV605 (CD44).

HuP3D primary cultures were developed using tissue biopsies and matching plasma from the same BCa patient (Figure 7a). Tissue biopsies were processed either into small organoids or as single cell suspensions. Cell proliferation of BCa cells detected as CD44+/EpCAM+ by both methodologies for the processing of fresh biopsies was not found to be significantly different with about a 2.5-fold and 3.3-fold growth compared to day 0 at days 3 and 7, respectively (Figure 7b and Figure S2). We further compared the feasibility of growing the same biopsy directly from fresh tissue or after a freeze/thaw cycle using the single cell suspension methodology. Cell proliferation of CD44+/EpCAM+ BCa cells from frozen conditions remained unaltered when compared to the cells from fresh tissue with about a 2.3-fold and 3.7-fold growth compared to day 0 at days 3 and 7, respectively (Figure 7c). 

Successful growth of frozen biopsies allowed us to further select three snap frozen biopsies from the Sanford biobank with a known clinical outcome after treatment with the same chemotherapeutic regimen. Plasma and biopsies from each of these patients were used in a precision-based approach and tested with the same chemotherapeutic regimen (Arimidex) as was utilized in the clinic after biopsy collection. The clinical effective concentration for Arimidex in patients was found in literature to be at 13.7 μg/L (45 µM) [40]. The Css (45 µM) and 1/3 (15 µM) of the Css were the concentrations tested on these patient tumor biopsies that, in the clinical setting, received Arimidex treatment following biopsy and had the clinical outcome after that treatment reported as response to treatment or resistance. Survival of EpCAM+ BCa cells after Arimidex treatment correlated with the reported clinical outcome. While EpCAM+ BCa cells from patient SH-8 with the “resistance” clinical outcome clearly revealed little to no effect of Arimidex at 45 µM, EpCAM+ BCa cells decreased to 49 and 17% for patients with moderate and strong responses to treatment at Css (45 µM) concentrations, respectively (Figure 7d). These results highlighted the feasibility of the precision-based capabilities of HuP3D cultures for the prediction of therapeutic efficacy. Finally, Figure 7e recapitulates the gating strategy used to detect BCa cells in 3D cultures where we were able to differentiate them from the other cellular TME components of the tumor biopsy by identifying BCa cells as CD45-/CD44+/EpCAM+ cells.

## 3. Discussion

The majority of BCa deaths are associated with recurrence or the spread of the disease [4,5]. When a patient is initially diagnosed, physicians have an arsenal of treatment regimens to choose from. However, a standard clinical treatment is used in BCa patients despite the fact that the BCa population is highly heterogeneous [6,7,8]. The main molecular subtypes of breast cancer are luminal A, luminal B, HER2 positive, and basal-like (triple negative). These intrinsic molecular subtypes have been associated with accurate predictions of recurrence and survival, especially for luminal tumors [41,42], and as predictors of therapeutic response [42,43]. The cellular crosstalk of a tumor and its TME is also a key contributor to clinical outcomes and responses in BCa patients [22,23,44,45,46,47]. Identifying patients who would respond well to specific drugs and those who may be most likely to exhibit resistance to them is a tremendous challenge. These features may explain why recurrence might lead to terminal outcomes and highlights a clear and urgent need for accessible models that can accurately predict responses to therapies for each individual patient prior to the development of recurrence.

Monolayer culture models have been widely employed to understand BCa over the past several decades, however, these 2D models still exhibit limited success in recreating the complexity of the disease [48,49]. Spheroids, organoids, 3D scaffolds, and PDX models are the most relevant alternative culture techniques. Although PDX results are often clinically relevant, to decrease animal distress, scientists are tasked to find alternative methods that replace, reduce, and refine the use of animals [50]. Consequently, other 3D cell-based assays are widely accepted and have proven to be versatile; they cut time, animal use, and cost, while facilitating more replicates and high-throughput endpoints [51,52,53,54,55,56]. Ideally, such platforms would include cancer-specific architectures, relevant biomechanical components, and cellular interactions (stroma-cancer and cell-extracellular matrix) found in patient tumors that assist in considering their inherent heterogeneity. Unlike other 3D culture models developed from exogenous materials such as Matrigel and other polymers [26,57,58,59], our 3D culture model is comprised of human blood plasma (patient-specific). In contrast to synthetic or not-patient derived ECMs, the use of a plasma-derived model provides a “blank slate matrix” where the cells have the ability to produce their highly specific ECMs and in turn avoid the incorporation of exogenous materials in the experimental models that might introduce undesired confounding artifacts to the recreation of tumor behaviors. Inclusion of artificial ECM components may affect the tissue stiffness of the artificial matrix, directly influencing drug accessibility or cellular behavior in in vitro screenings [60]. Of note, the use of human plasma (fibrinogen cross-linking to fibrin) as the matrix not only supports cell structure [61,62], but it also serves as a reservoir for nutrients, growth factors, cytokines, extracellular vesicles, and signaling molecules that help with the development of more personalized models [30,63,64,65,66]. Even though the composition of plasma proteins is not abundant in tumor tissue, cells in our plasma-matrix model enhanced the secretion of TME components relevant to the disease. We have previously shown that ECM proteins such as collagen and fibronectin are secreted by BCa cells in HuP3D cultures [66]. EV secretion was also enhanced in HuP3D when compared to 2D. It should be clarified that the differences between the number of particles/mL and the EV protein concentration are reflecting the measurement of any particle isolated by ultracentrifugation in comparison to the actual protein measurement. Enhanced secretion of EVs in 3D models has previously been reported [67]. Finally, human plasma was found to be an excellent reservoir for cytokines involved in key cancer hallmarks including: pro-inflammatory cytokines (IL-β1, MIP-1a, TNFα, and RANTES) [68,69], chemokines influencing the invasiveness and migration (SDF-1) [70], cytokines supporting tissue repair/ECM degradation and remodeling (TIMP1, TIMP2, MMP1, and MMP9) [71,72], and cytokines promoting cell growth (INF-γ, PDGF-AB, IL-2, and LIF) [69,73]. In addition, cytokines involved in fibrogenesis (EGF, IGF-I, HGF, and PDGF-AB) [74,75] were found in plasma from healthy subjects and BCa patients. CA125 is a common blood marker tested in breast cancer and ovarian cancer and it allows for the identification and distinction of the plasma from BCa patients [76,77]. Although the plasma fibrinogen level may be considered a possible marker for the clinical response and prognosis of patients with breast cancer [78], we were able to observe a slightly increased fibrinogen level in our BCa samples, but not enough to be significant in the samples used in our studies. This similarly allows for a more relevant comparison among the HuP3D cultures using either healthy or BCa patient plasma.

HuP3D chemical characterization allowed us to define the optimal cross-linking and stabilization conditions in order to further develop HuP3D cultures. Calcium chloride was selected as the recommended cross-linker component, even though all the other tested options yielded favorable outcomes. The main reasons for this decision were 1) it had the fastest cross-linking time to avoid cell settling at the bottom of the gel, 2) thrombin and factor XIII are enzymatic clotting factors while CaCl_2_ is an ionic compound that is readily available and naturally present during blood coagulation [79,80,81], and 3) price. Cross-linking time and cross-linking concentrations can affect the mechanical properties of the HuP3D cultures, in turn affecting cellular response [82,83]. Further characterization of the additional cross-linkers not used in HuP3D cultures would be needed in order to determine the possible implications on cellular responses for those components. Optimization of the cross-linker concentration is fundamental. As an example, increased concentrations of CaCl_2_ higher than the recommended dose revealed a less efficacious cross-linking time. These results could be explained by the presence of free calcium ions after reaching optimal plateau concentrations which could affect fibrinogen cross-linking capacity [84]. AMCHA was selected as the suggested antifibrinolytic based on its capacity to induce the highest weight gain in the matrix when compared to the other antifibrinolytics. 

Furthermore, the aspiration for the development of model systems for cancer modeling that more closely resemble human physiology is currently an area of high emphasis. We hypothesized that HuP3D cultures would support the growth and expansion of primary breast cancer tumors, as well as allow for the screening of cancer drugs for further prediction of clinical therapeutic efficacy. While many studies have shown the crucial role of the TME in tumor progression in BCa [85,86,87], surprisingly few have focused on the comparison of the HME and TME. This importance of malignant versus healthy microenvironment on tumor progression has been successfully demonstrated in other types of cancer [88]. Additionally, cellular function can be altered by growth on 3D substrates and these alterations can be dependent on the 3D membrane concentration [89]. To date, there are no good quantitative models which allow for the deep characterization and experimental manipulation of the role of the TME in tumor progression. Our technology provides the basis to address these problems. HuP3D cultures confirmed the significant role of the TME in cancer progression in BCa cell lines, revealed through cell survival signaling pathways (pAKT and Ki67), while apoptosis pathways remained unaltered (cleaved caspase 3) (Figure 2). Some cell lines did not show an increase in cell proliferation rate at day 7 compared to day 3 in either mono-cultures or when co-cultured with HME. These results could be explained by biological changes that occur in 3D cultures including, but not limited to, changes in cell polarization and differentiation [90] and changes in cytoskeletal elements and extracellular matrix proteins secretion or deposition [91] over the duration of the proliferation time period. It is important to emphasize that primary BCa cultures in HuP3D models contained BCa cells and all the accessory TME cellular components from the original biopsy, recapitulating the in vivo environment of the BCa cells in a 3D culture with plasma containing key signaling molecules from the same patient. In addition, HuP3D cultures demonstrated two successful methodologies to grow primary patient material as well as confirming consistent growth from fresh or frozen biopsies. We found the single cell suspension methodology appealing when sorting, and the separation of a distinct cell population was of interest for the downstream analysis. However, organoids allowed us to preserve the original tumor architecture, which could heavily affect progression and drug resistance [92,93,94]. These results revealed a great opportunity for open collaborations, as biopsies can be frozen and preserved for future use in our studies, so we can receive samples from collaborators in order to perform custom screenings, and we have two successful techniques for material growth in the case of minimal initial tumor biopsy material. Common problems in biopsy collection that could impact future studies include the method of sample preservation and processing, tissue quality, the amount of tumor contained within the tissue collected (especially in pre-chemotherapy groups), as well as heterogeneity amongst the samples from each patient’s tumor [95].

The IC_50_ is the most commonly reported drug response metric [96], but unfortunately, in many cases, it has been used incorrectly. Traditionally used drug response metrics, such as IC_50_ or EC_50_, do not take into account the initial cell population (γ_0_) and the number of cell divisions during the course of the assay, which can vary amongst different cell lines, due to the fact that they depend on cell density, media composition, and experiment duration [39]. In our studies, to overcome these limitations of traditionally used drug response metrics based on relative cell count, we also determined growth rate (GR) metrics. GR parameters are more suitable to be calculated in our drug response assays because they consider the initial cell population and the differences in the growth rates among the BCa cell lines in the HuP3D cultures. Our studies looked at whether differences in cell growth rates of cancer cells in the HuP3D and a wide variety of drug metrics could radically impact drug responses, leading to an incomplete picture when predicting drug efficacies. We detected a significant heterogeneity among the different BCa cell lines, drugs, and drug response metrics, suggesting the need for the use of more than one type of drug response metric to predict drug efficacy and the requirement of a method for personalized prediction of therapeutic response. Relevant examples of the importance of including different metrics to measure drug effect include, for example, the effect of docetaxel on MDA-MB-231 and ZR-75-1. While looking at relative cell count, docetaxel was cytotoxic for MDA-MB-231 and cytostatic for ZR-75-1, but on the GR value curves the responses are inverted with a cytotoxic effect for ZR-75-1 and a cytostatic effect for MDA-MB-231. The incorporation of the initial cell population and the differences in the growth rate of these cell lines did indeed affect the initial assumptions of response [37,39]. Some of the described metrics showed higher variation between biological replicates. These results could be explained by underlying biological mechanisms or by the standardization of assay methodology. Automated plating and dosing will help to increase consistency, precision, and reproducibility [97]. Drug response metrics based on relative cell count could be influenced by the number of cell divisions or plating density as these metrics do not take into account the initial cell population and the number of cell divisions during the course of the assay [37,39]. GR metrics could be influenced by the potency and efficacy of the drug and resistance profiles of the cell lines [29], as well as by the temporal dependence of drug response [97]. In addition, both types of metrics could be affected by the intra-assay variability due to the fact that the matrix is generated from plasma from non-matching subjects. Plasma could be pooled in batches to attenuate this effect in cell lines where matching plasma is not available. Additionally, further studies will look into combination therapies of chemotherapeutic drugs, natural compounds, or monoclonal antibodies in order to provide more effective strategies for treating BCa due to the resistance of BCa cells and the various side effects of current treatments [98,99]. Drug efflux mechanisms will be further evaluated in HuP3D cultures, due to the fact that an elevated efflux of anticancer agents, leading to decreased intracellular drug accumulation, has been considered to be the major reason for chemotherapy resistance [99,100]. Further studies should also evaluate how cancer cells in 3D coordinate their activity to respond to drugs at different stages of their growth and development. Furthermore, the comparison of the drug response metrics from our HuP3D model to the IC_50_ values obtained from literature search metrics for 2D or other 3D models, with effective chemotherapeutic concentrations in phase I or II clinical trials patients (Css), revealed a very poor, positive correlation between Css and 2D models and a moderate correlation with other 3D models. Of note, the other 3D models group included PDX, spheroids, organoids, and 3D scaffolds as a combinatory group in order to obtain enough significant information from published literature for the seven drugs to establish Pearson correlations. We acknowledge that it will have an added value to compare to each of them separately, but it was complicated to gather enough references for each group for all the drugs. The metrics E_inf_ and GEC_50_ did not correlate well with Css or with any of the other HuP3D metrics. A particular problem with GEC_50_ is that this metric is relevant for drugs that have poor potency and that do not reach a GR value below 0.5. If the fit of the curve is not significantly better than that of a flat curve, the parameter is set to 0 [37,39]. Furthermore, our data showed a big variability for MCF7 GEC_50_ values. Some problems also appear with E_inf_ where, when the dose response curves reach a plateau at the highest tested concentrations, the value for E_inf_ is similar to E_max_. E_max_ depends on the concentration range used in the assay, thus, both metrics should be used with caution when comparing them to other studies using a different concentration range [37]. On the other hand, a significant and strong correlation with clinical effective concentrations was found for eight of the HuP3D metrics (seven positively correlated and one negatively correlated). Additionally, there was a very weak correlation between HuP3D metrics and 2D models and a moderate correlation to other 3D models, revealing similar profiles as those for HuP3D and Css. These results just confirm that the 2D cell culture models cannot efficiently predict the therapeutic response and the effective drug dose in cancer patients, due to their inability to represent the TME heterogeneity. Despite a good response in 2D tissue culture and PDX models, many patient tumors have shown a chemoresistance profile [101]. There have been a good number of drug response studies done that compare the 3D models with monolayer cell cultures and their clinical outcomes, and all of them conclude that 3D models are more suitable to predict drug responses in a precision-based manner in accordance with clinical effective concentrations [48,49,58,102,103]. Dhiman et al. carried out inhibition studies of different concentrations of tamoxifen for six days in MCF7 monolayer and 3D chitosan cell cultures and demonstrated that 3D models exhibited an increased resistance to tamoxifen when compared to 2D cell cultures, and a higher dose of the drug was needed to achieve a comparable cytostatic effect in 3D [104]. Imamura et al. compared the growth rate of 2D and 3D models using BCa cell lines and patient-derived xenografts in the presence of paclitaxel, doxorubicin, and 5-fluoro-uracil at concentrations of 0.1, 1, and 10x to the AUC values obtained in clinical pharmacokinetic studies and saw that 3D cell culture models are a significantly better choice in simulating important tumor characteristics and drug resistance [48]. Hongisto et al. compared 63 drug responses in JIMT1 BCa cells grown in monolayer, 3D poly (2-hydroxyethyl methacrylate), and 3D Matrigel cell culture models and observed high discrepancies between the models, where the Matrigel 3D model had significantly higher drug sensitivity than the others, suggesting that 3D cultures are a better alternative to traditional 2D cell cultures and can provide a platform for high-throughput drug screening. The composition, however, of the 3D model influences the drug response [103]. Breslin and Driscoli reported drug response assays with docetaxel and neratinib in HER2-positive BCa cell lines in 2D and 3D poly-HEMA models and found that 3D models are more drug resistant than monolayer cell cultures, which could be facilitated by upregulating drug transporters and receptor proteins and metabolizing enzyme activity. 

All in all, there are some limitations when it comes to the use of a comparative analysis between the drug response metrics from literature searches of different cell culture models and actual studies. Haibe-Kains et al. compared the IC_50_ and the AUC of 15 drugs in 2D cultures of 471 cell lines which were reported in two large-scale pharmacogenomics (Cancer Genome Project and Cancer Cell line Encyclopedia) and consisted of vast discrepancies. Only one drug had a moderate Spearman correlation coefficient in the measured response (r_s_ = 0.61) and one had a relatively fair correlation (r_s_ = 0,53), while the IC_50_ could not be estimated in many cases [105]. Furthermore, there are clear differences between the cell growth rates in 2D and 3D platforms that make it difficult to compare the drug response metrics across them and even raises the question whether or not the same reporting tool should be used. Brooks et al. reviewed 25 publications of 2D and 3D chemotherapeutic drug screening assays and found many difficulties that can affect drug metrics comparisons [29]. Some studies reported an IC_50_ value despite the fact that the drug was not effective enough to reduce the cell population by half. In some literature cases, they noticed that the EC_50_ and GI_50_ were confused for the IC_50_, which was interpreted in the context of growth inhibition. Moreover, they identified that in the highly potent drugs, the IC_50_ does not differ much between publications, however, when the drug sensitivity is moderate or low, IC_50_ values tend to be extremely variable in 2D and even more remarkably variable in 3D culture models. All these discrepancies are formed because the drug response metric values may depend on the experiment protocol (medium, cell type, time of incubation, etc.), the composition of 3D models (stromal cells, ECM, different biomaterials, etc.), type or length of the assay, and the method of analysis (MTT, flow cytometry, ATP, etc.). Furthermore, it is important to bear in mind that researchers can use different forms of a nonlinear regression equation to calculate the drug response metrics and utilize different methods to handle problems such as outliers. Additionally, there are some parameters that cannot always be calculated due to the shape of the fitted curve (IC_50_, GEC_50_) or when the cells do not grow exponentially (GR_50_), and some of them should be used carefully in a comparison analysis between assays with a different concentration range (E_max_ and AUC) [29]. Despite all these limitations, our results showed the feasibility and efficacy of the HuP3D drug response metrics to predict clinically effective therapies better than current preclinical models. Our model is also compatible with high-content imaging and high-throughput applications. However, despite the fact that 3D cultured cancer cell lines can reflect better physiological features of the cells than conventional 2D models, the forced 3D architecture incorporating cancer cell lines does not recapitulate the original tumor characteristics and cell-cell interactions with accessory tumor components. For that reason, we believe that the growth of primary tumors for use in drug screening using our model will better reflect the physiological features of an actual patient’s cancer and better predict the clinical efficacy. 

The field of “personalized” medicine is a burgeoning area of research where the clinical use of such “precision” or “targeted” treatment approaches in the role of oncology are growing rapidly [104,106,107]. Traditionally, such capabilities have been limited to in vitro for PDX, greatly limiting the treatment options for cancer patients in a short-time therapeutic window. HuP3D culture drug screening assays gave therapeutic responses in only one week, while the PDX models will take months. Using a small cohort of BCa patients with very distinct responses to the same chemotherapeutic treatment, we were able to retrospectively predict the same clinical outcomes detected in the clinical setting. These results emphasize the unique capabilities of HuP3D cultures as a reproducible, human-derived, high-throughput in vitro culture model for demonstrating drug sensitivity and clinical efficacy. Employment of a high-throughput human model could provide enormous cost savings in clinical studies by validating therapies in improved preclinical models [108,109,110]. However, much work needs to be done, as our understanding of the potential efficacy of targeted treatments remains quite limited but continues to grow. It has become clear that the identification of a particular genomic alteration often does not correlate with clinical efficacy when an agent “targeting” that alteration is utilized [111,112,113]. We will further investigate the efficacy of novel treatment options in a focused fashion utilizing HuP3D cultures, which we hypothesize will be a more accurate representation of in vivo activity of these novel targeted agents. This will expand our understanding of the underlying biology of this malignancy, help guide our understanding as to the potential benefit of novel agents, and perhaps eventually allow us to perform patient-specific drug sensitivity testing to guide patient care if this model is further validated in a prospective setting. This goal fits seamlessly into our mission to advance our knowledge of cancer and to provide novel and effective treatment options for our BCa patients. Personalized prediction of therapeutic efficacy will directly address the overarching challenge existing in BCa of preventing the mortality associated with recurrence of the disease by providing the ability to predict therapeutic efficacy in each BCa patient case with a more targeted, effective, and less toxic treatment regimen, saving not only medical costs but also women’s lives. 

In summary, HuP3D efficiently supported the growth and expansion of BCa cell lines and primary breast cancer tumors. Significant and strong correlations between clinical effective concentrations in patients were found for eight out of ten metrics for HuP3D, revealing the HuP3D culture model as a feasible and efficacious platform for supporting the growth and expansion of BCa, allowing high throughput drug screening and predicting clinically effective therapies better than current preclinical models.

## 4. Materials and Methods 

### 4.1. Reagents

Type I collagenase, trans-4-(aminomethyl)cyclohexanecarboxylic acid (AMCHA), thrombin, factor XIII, aprotinin, calcium chloride (CaCl_2_), epsilon-aminocaproic acid (EACA), 4-aminomethylbenzoic acid (PAMBA), and dimethyl sulfoxide (DMSO) were purchased from Sigma-Aldrich (Saint Louis, MO, USA). Fluorescent cell trackers such as 3,3′-dioctadecyloxacarbocyanine perchlorate (DiO, excitation, 488 nm; emission 525/50 nm), 1,1′-Dioctadecyl-3,3,3′,3′-tetramethylindodicarbocyanine perchlorate (DiD, excitation, 635 nm; emission, 655–730 nm), and DiIC18(7) (1,1′-dioctadecyl-3,3,3′,3′-tetramethylindotricarbocyanine iodide) (DiR, excitation, 750 nm; emission, 780 nm) were purchased from Invitrogen (Carlsbad, CA, USA). Drug treatments for cell line studies including methotrexate (MTX), paclitaxel (PTX), capecitabine (CAP), cyclophosphamide monohydrate (CYCLO), carboplatin (CARBO), epirubicin hydrochloride (EPI), and docetaxel (DTX) were purchased from MedKoo (Morrisville, NC, USA). Drug treatment of patient material using Anastrazole (Arimidex) was purchased from Selleck Chemicals (Houston, TX, USA).

### 4.2. Cell Lines

Breast cancer (BCa) cell lines representing different molecular subtypes (luminal A: MCF7, ZR-75-1, HER2: MDA-MB-453, SK-BR-3 and triple negative: MDA-MB-231) (see Table 1) were used for experiments. All cells were cultured at 37 °C, 5% CO_2_; ZR-75-1 and SK-BR-3 cells in RPMI-1640 media with L-glutamine (Corning CellGro, Mediatech, Manassas, VA, USA) and MCF7, MDA-MB-453, and MDA-MB-231 in DMEM media with L-glutamine, 4.5 g/L glucose and sodium pyruvate (Corning CellGro, Mediatech, Manassas, VA, USA) supplemented with 10% (V/V) fetal bovine serum (FBS, Gibco, Life Technologies, Grand Island, NY, USA), 100 U/mL penicillin, and 100 μg/mL streptomycin (Corning CellGro, Mediatech, Manassas, VA, USA). Labeling of BCa cells (1 × 10^6^ cell/mL) was done prior to experiments with DiO (10 µg/mL), DiD (10 µg/mL), or DiR (10 µg/mL) by incubation of cells with the surface marker for 1 h.

### 4.3. Human Samples

Tissue biopsies were obtained from BCa cancer patients undergoing a tumor biopsy and healthy subjects undergoing breast surgery from the Sanford USD Medical Center, Edith Sanford Breast Cancer Center and Sanford Biobank in Sioux Falls, SD. Healthy subjects are defined as patients with abnormal growths or other deviations in the breast tissue determined by the pathologists to be normal or benign, non-cancerous tissue. Informed consent was obtained from all subjects with approval from the Sanford Health Institutional Review Board and in accordance with the Declaration of Helsinki. Tissue biopsies were weighed, pre-washed, and minced into pieces approximately 0.2 mm^2^ with a sterile scalpel and forceps. Minced tissue biopsies were enzymatically dissociated in dissociation buffer (0.1% *w/v* type I collagenase and 3mM CaCl_2_ solution), using a guideline of 1 mL dissociation buffer per 100 mg tissue, followed by sequential filtration for the generation of small organoids and single cell suspensions. Small organoids were directly grown in culture or stored frozen for further analysis. Single cell suspensions were counted. Single primary cell suspensions from healthy breast tissue were further identified as a healthy microenvironment (HME). Primary CD44+ and CD44- cells (identified as the tumor microenvironment (TME) cellular component) were sorted from the digested BCa patient biopsies using magnetic-bead sorting with Miltenyi beads, following manufacturer instructions. Single cell suspensions were then either grown in culture or stored frozen for further analysis. Peripheral blood from BCa patients or healthy subjects was obtained using venipuncture and collected in whole blood collection tubes (BD Lavender K2-EDTA Vacutainer, except for fibrinogen assay samples in which blood was collected in the BD Blue Sodium Citrate Vacutainer). Plasma was separated using centrifugation at 800 × *g* for 10 min; then, plasma was subjected to a secondary spin at 400× *g* for 10 min, immediately aliquoted, and stored at −80 °C. 

### 4.4. Development and Characterization of HuP3D Cultures

HuP3D cultures are formed through the cross-linking of fibrinogen found naturally in plasma (Figure 1a). Cross-linking time was assessed by measuring the time necessary to achieve matrix cross-linking using three relevant cross-linkers of the blood coagulation process [79,80,114] including thrombin (0–5 mg/mL), CaCl_2_ (0–5 mg/mL), and factor XIII (0–6 mg/mL) (Figure 1c). The stabilization effects of preventing fibrin degradation and stability improvement in the scaffold were assessed by surveying several chemical antifibrinolytic agents including AMCHA (0–10 mg/mL), aprotinin (0–550 mg/mL), EACA (0–2.5 mg/mL), and PAMBA (0–2.5 mg/mL). The stability of the scaffold was studied by measuring each scaffold weight at day 0 and again measuring scaffold weight at the conclusion of a three-week time period (Figure 1d). CaCl_2_ and AMCHA were selected as the recommended components for all future studies. After initial HuP3D characterization, the following methodology was utilized for the remaining experiments: a mixture of plasma, primary cell suspension (3–5 × 10^4^ cells/scaffold) in RPMI-1640 or DMEM complete media, AMCHA, and CaCl_2_ was prepared with a 4:4:1:1 volume ratio, respectively. In order to avoid intra-assay variability due to the matrix, plasma was pooled in batches. HuP3D scaffolds were allowed to incubate at 37 °C with 5% CO_2_ to cross-link in a 96-well plate or 8-well chambered slide for 3 h before the addition of completed DMEM or RPMI-1640 media on top of the scaffolds to prevent drying. Media was changed every 2–3 days for the duration of each experiment.

HuP3D culture scaffold structure and morphology was analyzed with scanning electron microscopy (SEM) using a FEI Quanta 450 scanning electron microscope at multiple magnifications as well confocal microscopy using a Nikon Ti2-A1TR confocal microscope (10× dry objective, 2.5× magnified) with images being analyzed using NIS Elements software (Nikon Instruments, Melville, NY, USA).

### 4.5. Fibrinogen Content in Plasma

Plasma from BCa patients and healthy subjects was sent to Sanford Laboratories where the fibrinogen content of each sample was determined through the clotting method of Clauss [115]. Clauss fibrinogen assay is a quantitative, clot-based, functional assay. The assay measures the ability of fibrinogen to form a fibrin clot after being exposed to a high concentration of purified thrombin. Briefly, plasma samples were loaded into the STA-R Evolution Expert Series Hemostasis System (Diagnostica Stago Inc., Parsippany, NJ, USA), and automated testing was carried out by the analyzer. Control reagents were prepared and run to confirm accurate and reproducible results. 

### 4.6. Cytokine Expression in HuP3D Cultures

The effect of cytokines contributed by healthy and cancerous plasma used in the HuP3D model was tested using a custom cytokine antibody array. Acellular HuP3D cultures were created with either plasma from a healthy subject or plasma from a BCa patient using serum-free media following the recipe previously described. After chemical cross-linking and stabilization was complete, the cultures were disrupted with a lysis buffer (created by combining RIPA buffer, PMSF (1:10), DMOG (1:10), DTT (1:5), phosphatase cocktail 2 and 3 (1:100)) and sonication. HuP3D culture supernatants were collected and analyzed using a C-Series Custom Cytokine Antibody Array (RayBiotech Inc., Norcross, GA, USA), according to the instructions provided by the manufacturer. The custom cytokine array includes the following cytokines: interleukin beta 1 (IL-β1), macrophage inflammatory protein 1 alpha (MIP-1a), tumor necrosis factor alpha (TNF-α), regulated upon activation, normal t cell expressed and presumably secreted (RANTES), CA125, epidermal growth factor (EGF), insulin-like growth factor 1 (IGF-1), hepatocyte growth factor (HGF), platelet-derived growth factor AB (PDGF-AB), interferon gamma (INF-γ), interleukin-2 (IL-2), stromal derived factor 1 (SDF-1), leukemia inhibitor factor (LIF), tissue inhibitor of metalloproteinase 1 (TIMP-1), tissue inhibitor of metalloproteinase 2 (TIMP-2), matrix metallopeptidase 1 (MMP-1), and matrix metallopeptidase 9 (MMP-9). Images of the chemiluminescence signals of each of the membranes were captured using a LI-COR Odyssey (LI-COR Biosciences, Lincoln, NE, USA) device with a 2 min exposure time. The chemiluminescence signal intensity of each spot was quantified using densitometric analysis (VisionWorks Software, Upland, CA, USA). Values for each cytokine were established by initially subtracting negative controls and then normalizing to positive controls for each of the membranes. Complete scanned array is included in Figure S4.

### 4.7. Proliferation Analysis by Flow Cytometry of BCa Cell Lines

The five breast cancer (BCa) cell lines were previously labeled with DiO and incorporated in HuP3D cultures either alone, in co-culture with HME (2.5 × 10^4^ cells/scaffold), or in co-culture with TME (2.5 × 10^4^ cells/scaffold). These cultures were grown and analyzed at days 0.5 (γ_0_), 3, and 7. On each day of analysis, HuP3D cultures were enzymatically digested with type I collagenase at a concentration of 20 mg/mL for 2–3 h at 37 °C. After 2–3 h of incubation, samples were prepared in PBS for flow cytometry by adding counting beads (424902, Biolegend, CA, USA) in addition to Sytox blue dead cell stain (excitation 358 nm; emission 461 nm) (S34857, Thermo Fisher Scientific, MA, USA) for viability to each sample. BCa cells were identified by gating live cells with a DiO+ signal using the FITC channel on the BD FACS LSRFortessa SORP (BD Biosciences, San Jose, CA, USA). A minimum of 5 × 10^3^ events was acquired per sample and the FACSDiva v.6.1.2 software was used to collect and interpret data. BCa cell counts were acquired and data was analyzed using FlowJo v10 (BD Lifesciences, Ashland, OR, USA). Data was normalized to a predetermined number of counting beads, and the proliferation of each condition (fold of γ_0_) was calculated and compared.

### 4.8. Cell Proliferation and Apoptosis Signaling

The breast cancer (BCa) cell lines (MDA-MB-231 and MCF7) were previously labeled with DiR and incorporated in HuP3D cultures either alone, in co-culture with HME, or in co-culture with TME. These cultures were grown and analyzed at day 7. HuP3D cultures were enzymatically digested, and isolated BCa cells were fixed and permeabilized (Fixation Buffer: 420801, Biolegend, CA, USA; 10× Intracellular Staining Permeabilization Wash Buffer; 421002, Biolegend, CA, USA). Cells were then stained with V450 conjugated anti-Cleaved Caspase 3 (560627, BD Biosciences, San Jose, CA, USA) and AlexaFluor488 conjugated anti-pAKT (560404, BD Biosciences, San Jose, CA, USA) and analyzed using flow cytometry. Fluorescence minus one (FMOs) samples were used as controls. BCa cells were identified by gating cells with a DiR+ signal; then, the mean fluorescence intensity (MFI) of the cleaved caspase 3 and pAKT signals was calculated for the BCa-DiR+ cell population. 

### 4.9. Immunohistochemistry Studies (IHC)

HuP3D cultures containing BCa cell lines were processed as previously described [66,116]. Specifically, HuP3D cultures were fixed in 10% neutral buffered formalin and processed on a Leica 300 ASP tissue processor (Leica Microsystems, Buffalo Grove, IL, USA). Gel shrinkage can occur during this processing. Paraffin-embedded 3D matrix sections were longitudinally sliced at 10 μm. The BenchMark ® XT automated slide staining system (Ventana Medical Systems, Inc., Oro Valley, AZ, USA) was used for antibody optimization and staining. The antigen retrieval step was performed using Ventana CC1 solution, which is a basic pH Tris-based buffer. Both primary and secondary antibodies were prepared in a 1X permeabilization buffer (BioLegend, San Diego, CA, USA). A Ventana iView DAB detection kit was used as the chromogen, and the slides were counterstained with hematoxylin. Anti-Ki-67 (CRM325, 1:100, Biocare Medical, Pacheco, CA, USA), and anti-cleaved caspase 3 (CRM229, 1:100, Biocare Medical, Pacheco, CA, USA) primary antibodies were used. The omission of the primary antibody served as negative control. Secondary antibodies used were biotin-conjugated goat anti-rabbit IgG (111-065-144, 1:1000, Jackson ImmunoResearch, PA, USA) and biotin-conjugated donkey anti-mouse IgG (715-065-151, 1:1000, Jackson ImmunoResearch, PA, USA), respectively. IHC images were imaged using an Aperio VERSA Bright field Fluorescence & FISH Digital Pathology Scanner (Leica Biosystems, Buffalo Grove, IL, USA).

### 4.10. Confocal Imaging and Analysis

Growth and dissemination of BCa cells-DiO within the HuP3D scaffolds was observed using confocal microscopy at day 3 and day 7. The HuP3D structure was formed in an 8-well Thermo Scientific™ Nunc™ Lab-Tek™ II Chambered Coverglass with a No. 1.5 borosilicate glass bottom and covered with DMEM or RPMI-1640 media. The culture tray was imaged using a Nikon Ti2-A1TR confocal microscope with a 10× objective lens (Nikon Instruments, Melville, NY, USA). Culture cells were exposed to 488 nm (DiO) excitation, and the light emissions at 500–530 nm were collected as a z-stack image of each scaffold with a depth of roughly 0.5 mm to 1 mm using a step size of 2 µm. The frame size of the image was 512 × 512 pixels, which was taken at a rate equivalent to 1 µs/pixel. For analysis, the cells within each of the HuP3D cultures were measured using various multi-step automated modules within the NIS Elements (Nikon Instruments, Melville, NY, USA) software. In the first step, a threshold using the GFP (DiO) channel was created to map the cells of interest. Briefly, a binary was created using the auto detect feature. To avoid bias, the smallest object was used to generate the binary via the auto detect feature. Values corresponding to different features of interest including EqDiameter, circularity, elongation, and mean intensity were obtained. To determine clustering, binary restrictions were employed for each parameter that ranged between the largest observed value as the upper confidence interval and 50% higher than the calculated mean value as the lower confidence interval. The number of objects inside this chosen range were selected as positive clusters. For each condition, all detected cells were analyzed per experiment.

### 4.11. Extracellular Vesical Isolation and Corresponding Vesical Characterization

Extracellular vesicles (EVs) were isolated and characterized in a similar manner to previously described methods [66,117]. In order to account for the presence of extracellular vesicles in plasma and the newly generated extracellular vesicles from cells in traditional 2D cultures compared to HuP3D cultures, we systematically isolated and characterized the EVs from plasma in culture media with no cells, plasma in 2D with cells, and plasma in HuP3D cultures with cells (MDA-MB-231 BCa cells, 1 × 10^6^ cells). RPMI-1640 media was supplemented with plasma instead of 10% FBS, and the plasma volume was adjusted to be equal in all conditions (HuP3D cultures contain plasma in the matrix as well as in the top cultured media). Cultures were grown for three days before the HuP3D cultures were enzymatically digested with collagenase and the liquid from all conditions was collected separately to purify and characterize the extracellular vesicle component. Extracellular vesicle purification was completed using differential ultracentrifugation starting with centrifuging the samples at 2500× *g* for 10 min in a Thermo Legend X1R centrifuge (Thermo Fisher Scientific, Waltham, MA, USA), twice, to pellet out cells. These cells were counted for further characterization of the extracellular vesicles. The supernatant collected after the first centrifugation was subjected to a second centrifugation step at 13,000× *g* for 30 min, twice, in the Sorvall RC+6 centrifuge using the F13–14 × 507 rotor (Thermo Fisher Scientific, Waltham, MA, USA),. The supernatant was again collected and finally the samples were exposed to a final centrifugation step at 117,000× *g* for 2 h using a WX80 Ultracentrifuge and a SureSpin 630/17 rotor (Thermo Fisher Scientific, Waltham, MA, USA),. Extracellular vesicle pellets were washed in PBS, and the previous centrifugation step was repeated. After the final spin, pellets were re-suspended in sterile PBS, and aliquots were frozen at −80 °C. Characterization of the extracellular vesicles was performed using a BCA protein assay kit (Thermo Fisher Scientific, Waltham, MA, USA) as well as dynamic light scattering (DLS) analysis using a Nanosight NS300 (Malvern Panalytical, Malvern WR14 1XZ, UK). Extracellular vesicle preparations were analyzed using the SpectraMax Plus 384 (Molecular Devices, San Jose, CA, USA) and SoftMax Pro software. Absorbance was measured at 562 nm, and protein concentration was estimated from the values using the BCA standard curve with a quartic model fit.

### 4.12. Dynamic Light Scattering (DLS) Analysis

Purified extracellular vesicles were characterized using dynamic light scattering (DLS) analysis with a NanoSight NS300 particle analyzer (Malvern Panalytical) with a blue 488 nm laser and the NanoSight NTA 3.3 software. 100 μL of each extracellular vesicle sample was diluted with filtered PBS at a ratio of 1:6 which produced between 3 and 100 particles/frame for analysis. Initial adjustments were made using the Auto Setup function and then manual fine tuning of the settings was completed to make extracellular vesicle particles clearly visible for analysis. A screen gain of 1–1.5 was selected and a camera level 10–14 was used depending on the sample to create the desired particle visibility. A standard operating procedure was setup to create five 60 s videos that were recorded using the Nanosight instrument, and the particle size and concentration values were analyzed using the Nanosight NTA 3.3 software at a screen gain of 10 and a detection threshold of 20. A NanoSight microliter OEM syringe pump module (Malvern Panalytical) was used to inject the sample using a syringe at a set flow rate of 100. 

### 4.13. Western Blot

Extracellular vesicle (EV) separation by mass was accomplished in polyacrylamide gel via SDS-PAGE, then transferred to a polyvinylidene difluoride membrane via a Bio-Rad Trans-Blot Turbo Transfer System, using the protocols established by the manufacturer. Membranes were then washed twice for 5 min each in TBST (10 mM Tris pH 8.0, 150 mM NaCl, 0.5% Tween 20). Next, membranes were incubated in 1:10 clear milk (ThermoFisher Scientific, Pierce Clear Milk Blocking Buffer, 1X) in TBST, then incubated with an antibody against CD9 (1:1000, Santa Cruz Biotechnology, sc-13118, Dallas, TX, USA) in a solution of 1:20 clear milk in TBST overnight (at least 12 h) at 4 °C. Membranes were washed three times for 10 min each in TBST, incubated in 1:20 clear milk in TBST twice for 30 min, then incubated with horseradish peroxidase (HRP) conjugated anti-mouse secondary antibody (1:3000, Santa Cruz Biotechnology, sc-516102) and HRP conjugated anti-biotin secondary antibody (1:3000, Cell Signaling, #7075, Danvers, MA, USA) in a solution of 1:20 clear milk in TBST for 1 h, protected from light. After this, the secondary antibody was removed and the membranes were washed three times for 10 min each in TBST. All washing and incubation steps were done at room temperature with agitation unless otherwise specified. Immobilon Forte Western HRP substrate was added to the membranes, and the membranes were imaged using a LI-COR Odyssey FC system (LI-COR Biosciences). Complete scanned gel for western blot shown in Figure 3d is included in Figure S5.

### 4.14. Drug Screening of BCa Cell Lines in HuP3D Cultures Analyzed by Flow Cytometry

The five breast cancer (BCa) cell lines were previously labeled with DiD and incorporated in HuP3D cultures. Half a day after plating, cells were treated with a DMSO control (γ_Ctrl_) and increasing concentrations 0.1 nM–300 µM of seven standard-of-care chemotherapeutic drugs including methotrexate (MTX), paclitaxel (PTX), capecitabine (CAP), cyclophosphamide monohydrate (CYCLO), carboplatin (CARBO), epirubicin hydrochloride (EPI), and docetaxel (DTX). Treatments were added on top of HuP3D cultures in order to simulate drug diffusion into a tumor. Treatments were refreshed at day 4, and BCa cells were retrieved from the different cultures for analysis at day 0.5 (γ_0_) and day 7. Samples were prepared in PBS for flow cytometry by adding counting beads (424902, Biolegend) in addition to Sytox green dead cell stain (excitation 504 nm, emission 523 nm) (S7020, Thermo Fisher Scientific, Waltham, MA, USA) for viability to each sample. BCa cells were identified by gating live cells with a DiD+ signal using the FL4 channel on a BD Accuri C6 instrument (CFlow Software, BD Biosciences, San Jose, CA, USA). A minimum of 5 × 10^3^ events was acquired per sample, and BCa cell counts were acquired and data was analyzed using the FlowJo v10 (BD Lifesciences, Ashland, OR, USA) software.

### 4.15. Determining Drug Metrics Using the GR Calculator 

GR calculator (http://www.grcalculator.org) was used to determine 10 drug treatment response metrics for the HuP3D cultures based on either relative cell count (IC_50_, EC_50_, E_max_, AUC, E_inf_) or cell growth rate (GR_50_, GEC_50_, GR_max_, GR_AOC_, GR_inf_) (Figure S3b). The IC_50_ is the most widely used metric in drug treatment assays, where it is used to measure the drug efficacy and represent the inhibitory drug concentration needed to reduce the cell population by half. EC_50_ is also an important metric that characterizes the effective concentration of the drug that causes half of the maximal response. E_max_ is the maximum effect of the drug or the number of viable cells at the highest drug dose used in the assay, and the AUC is the area under the viability curve which indicates the cumulative effect of the drug. E_inf_ indicates the drug effect at infinite drug concentration. The GR values show the partial inhibition effect of the drug when it achieves GR values from 0 to 1, with the cytostatic effect being represented when the value is equal to 0 and the cytotoxic effect being represented when it lies between 0 and −1. GR_50_ is the drug concentration that reduces the cell growth rate by half, however, it is required that the cells grow exponentially (γ_final_/γ_0_ >2) in order to determine this metric. GEC_50_ indicates the drug dose that causes half of the maximal effect on the cell growth, and GR_max_ is the maximum effect on growth rate from the highest tested drug concentration. GR_AOC_ is the area over the GR curve that represents the variation in drug efficacy and potency, and GRinf indicates the maximal effect on the growth rate caused by the drug at an infinite dose [29,39]. All the parameters were calculated following the provided GR calculator instructions, using cell lines and treatment types as grouping variables for analysis. Briefly, four replicates for each assay condition (cell line, drug, and drug concentration) were averaged and the relative cell count was determined as y_(c)_/y_ctrl_, where y_(c)_ and y_ctrl_ are the number of viable cells in the presence of the drug at concentration c or at the control DMSO concentration, respectively. All the required conditions (cell line, drug, concentration, cell count at concentration c, initial cell count without drug treatment, and control cell count with DMSO treatment) were loaded into the online GR calculator tool, where the above-mentioned drug response metrics were calculated and analyzed.

### 4.16. Correlation of Drug Metrics

To evaluate the association between different variables, correlation tests were performed using the ggpubr R package. The Pearson correlation (r) was assessed in order to measure the linear dependence between two variables after confirmation of a normal distribution of the data. Correlation coefficients were compiled as a variable ranging between −1 (red) and 1 (blue), indicating a strong negative and positive correlation, respectively. A dot plot visualization of the Pearson correlation and *p* value significance was also used to visually assess differences between HuP3D metrics when compared to 2D IC_50_, other 3D model IC_50_, and clinical Css values. A violin plot was performed to evaluate the distribution of the data relative to the metric and experiment type from a distribution standpoint. Finally, a correlogram with a significance level indicating the most correlated variables in HuP3D metrics was performed using the R corrplot package.

### 4.17. Proliferation and Drug Screening Analysis by Flow Cytometry of Primary BCa Cells 

Fresh or frozen small organoids and single cells (5 × 10^4^ cells/scaffold) obtained from BCa patient biopsies were incorporated in HuP3D cultures. These cultures were grown and analyzed at days 0.5 (γ_0_), 3, and 7. Three breast cancer patients with a known clinical outcome and treated with the same chemotherapeutic regimen were identified at the Sanford Biobank. Patient clinical follow-up was greater than two years and their response was categorized as resistance or response to treatment. Single cells were obtained from the frozen BCa biopsies as previously described. Single cells (5 × 10^4^ cells/scaffold) obtained from the BCa patient biopsies were incorporated in HuP3D cultures. These cultures were grown and treated with a DMSO control (γ_Ctrl_) and Arimidex concentrations of 15 µM (1/3 Css) and 45 µM (Css). Treatments were refreshed at day 4, and BCa cells were retrieved from the different cultures at day 7. On each day of analysis, HuP3D cultures were enzymatically digested and isolated cells were stained with FITC conjugated anti-CD45 (304038, Biolegend, San Diego, CA, USA), BV605 conjugated anti-CD44 (103047, Biolegend, San Diego, CA, USA), and PECy7 conjugated anti-EpCAM CD326 (324222, Biolegend, San Diego, CA, USA). Samples were prepared in PBS with 1% BSA (*w/v*%, Sigma-Aldrich, Saint Louis, MO, USA) for flow cytometry by adding counting beads (424902, Biolegend) in addition to Live/Dead Blue Cell Stain (L34962, Thermo Fisher Scientific, Waltham, MA, USA) for viability to each sample. BCa cells were identified by gating live cells as CD45-/CD44+/EpCAM+ cells on the BD FACS LSRFortessa SORP (BD Biosciences). A minimum of 5 × 10^3^ events was acquired per sample, and FACSDiva v.6.1.2 software was used to collect data. BCa cell counts were acquired and data was analyzed using FlowJo v10 (BD Lifesciences, Ashland, OR, USA). Data was normalized to a predetermined number of counting beads, and the proliferation of each condition (fold of γ_0_) and survival (% of control) was calculated and compared. 

### 4.18. Statistical Analysis of Data

Experiments were completed with replicates in triplicate (technical replicates) and repeated at least three times (biological replicates), unless noted otherwise in the figure legends. Results have been presented as a mean value ± a standard deviation value. Statistical significance was analyzed using Student’s t-test, one-way ANOVA, or two-way ANOVA; a *p* value less than 0.05 was considered significant. An interquartile range (IQR) was used to remove outliers.

## 5. Conclusions

Human plasma-derived 3D culture (HuP3D) is a physiological, patient-derived, tumor-like 3D culture method that supports the efficient growth and expansion of BCa cell lines and primary breast cancer tumors, as well as allowing for the screening of cancer drug responses in BCa cell lines and primary BCa tumors. A significant and strong correlation with the clinical effective concentration in patients was found for eight out of ten of the HuP3D metrics, revealing HuP3D cultures as a feasible and efficacious technique to predict clinically effective therapies better than current preclinical models.

## 6. Patents

Pilar de la Puente, Somshuvra Bhattacharya, and Kristin Calar have a provisional patent application on the method described in this manuscript.

## Figures and Tables

**Table 1:** Characteristics and molecular subtypes of the BCa cell lines used in this article.

Cell Line	Doubling Time	Origin	Pathology	Subtype	ER	PR	HER2
MDA-MB-231	38 h	Pleural Effusion	Adenocarcinoma	Triple Negative B	−	−	−
MDA-MB-453	38 h	Pleural Effusion	Adenocarcinoma	HER2 Positive	−	−	+
ZR-75-1	54 h	Ascites	Invasive Ductal Carcinoma	Luminal A	+	−/+	−
MCF7	48 h	Pleural Effusion	Invasive Ductal Carcinoma	Luminal A	+	+	−
SK-BR-3	30 h	Pleural Effusion	Adenocarcinoma	HER2 Positive	−	−	+

Doubling time designates the time required for the BCa cell line population to double in size in traditional 2D cultures. Origin and pathology columns show the clinical properties of each of the BCa cell lines and where each of the cell lines is derived from. Categorization of the BCa cell lines into molecular subtypes such as luminal A, luminal B, HER2 positive, and triple negative is based on the primary features of each cell line and is represented according to the positive or negative status of estrogen receptor (ER), progesterone receptor (PR) and human epidermal growth factor receptor 2 (HER2) characteristics.

## Supplementary Materials

The following are available online at https://www.mdpi.com/2072-6694/12/7/1722/s1, Figure S1: (a) Cell proliferation data of each of the independent BCa cell lines. (b) Quantification of confocal microscopy images. Quantified number of MDA-MB-231 cells detected growing within HuP3D cultures after 3 and 7 days and representative confocal images reflecting the selection pane used via binary editor (DiO tagged green cancer cells are highlighted red when selected for counting considerations). (c) Quantified number of MDA-MB-231 cell clusters only in scanned z-stack within HuP3D cultures after 3 and 7 days and representative confocal images reflecting the selection pane used via binary editor for counting MDA-MB-231 cell clusters (clusters of DiO tagged green cancer cells are highlighted red when selected for counting considerations), (*) *p* < 0.05; Figure S2: Micrographs of the 5 BCa cell lines and primary cells (single cells and organoids) growing in HuP3D cultures at day 3. Scale Bar = 100 µm; Figure S3: HuP3D high-throughput drug screening. (a) BCa cell lines were previously labeled with DiD and incorporated in HuP3D cultures. After matrix stabilization, cells were treated with a DMSO control (γ_Ctrl_) and increasing concentrations 0.1 nM–300 µM of seven standard-of-care chemotherapeutic drugs. Media was refreshed at day 4, and HuP3D cultures were enzymatically digested to be analyzed using flow cytometry at day 0 (γ_0_) and day 7. Data was analyzed utilizing the GR calculator to determine 10 drug response metrics. (b) Cytotoxic and cytostatic effects of HuP3D drug treatments depicting the HuP3D culture drug metrics from the relative cell count curve including the half maximum inhibitory concentration (IC_50_), maximal measured efficacy (E_max_), area under the curve (AUC), half maximal response concentration (EC_50_), and the effect of the drug concentration at infinite concentration (E_inf_), as well as from the GR curve including the half maximum growth rate inhibition concentration (GR_50_), the maximum effect of the drug at the highest tested concentration (GR_max_), area over the curve (GR_AOC_), the half maximal growth rate inhibition response concentration (GEC_50_), and the effect of the drug at infinite concentration (E_inf_); Figure S4: Complete custom cytokine array image; Figure S5: Complete scanned gel for western blot shown in Figure 3d; Table S1: HuP3D culture drug metrics from four independent assays, average and standard deviation, and references from 2D models, other 3D models, and Css clinical effective drug concentrations.
